# Supplementary material for: Predicting visual function by interpreting a neuronal wiring diagram
Source: Nature. 2024 Oct 2;634(8032):113–23. doi: 10.1038/s41586-024-07953-5 (PMC11446822; doi:10.1038/s41586-024-07953-5)
Supplement: Supplementary file 6 — CRF and ERF predictions for individual TmY4 and TmY9 cells. Analogous to Supplementary Data 3, but for TmY target types. Shown are the top four monosynaptic pathways, the strongest pathway passing through each of the top ten intermediary types (ranking from Extended Data Fig. 7), and the trisynaptic pathway Tm1–TmY–Dm3–TmY (see the section entitled Prediction of spatial normalization). [file 41586_2024_7953_MOESM6_ESM.zip › DataS4/TmY9qΓèÑ/720575940637219391.pdf]

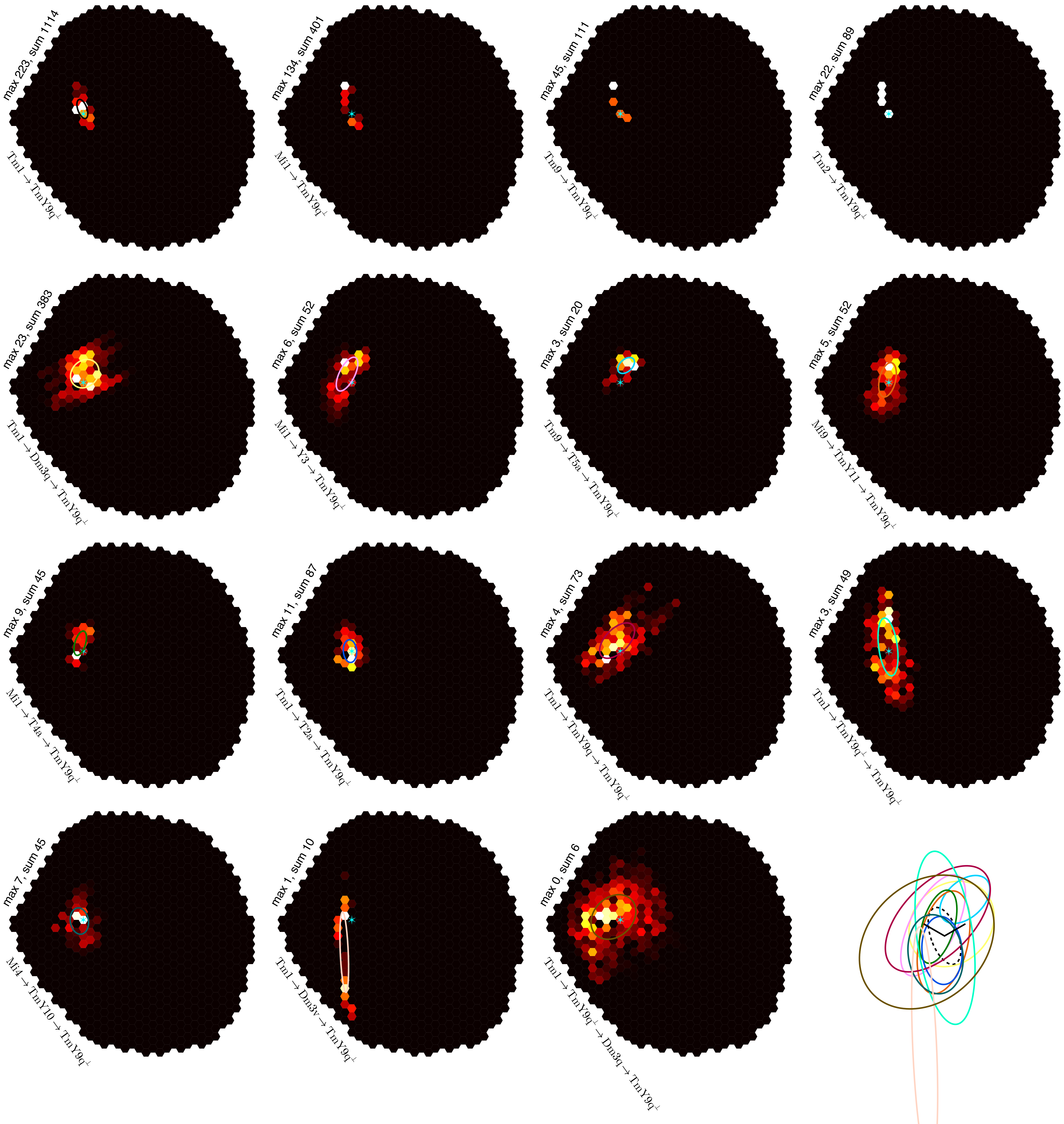

max 223, sum 1114
Tm1 → TmY9q$^{\perp}$
max 134, sum 401
Mi1 → TmY9q$^{\perp}$
max 45, sum 111
Tm9 → TmY9q$^{\perp}$
max 22, sum 89
Tm2 → TmY9q$^{\perp}$
max 23, sum 383
Tm1 → Dm3q → TmY9q$^{\perp}$
max 6, sum 52
Mi1 → Y3 → TmY9q$^{\perp}$
max 3, sum 20
Tm9 → T5a → TmY9q$^{\perp}$
max 5, sum 52
Mi9 → TmY11 → TmY9q$^{\perp}$
max 9, sum 45
Mi1 → T4a → TmY9q$^{\perp}$
max 11, sum 87
Tm1 → T2a → TmY9q$^{\perp}$
max 4, sum 73
Tm1 → TmY9q → TmY9q$^{\perp}$
max 3, sum 49
Tm1 → TmY9q$^{\perp}$ → TmY9q$^{\perp}$
max 7, sum 45
Mi4 → TmY10 → TmY9q$^{\perp}$
max 1, sum 10
Tm1 → Dm3v → TmY9q$^{\perp}$
max 0, sum 6
Tm1 → TmY9q$^{\perp}$ → Dm3q → TmY9q$^{\perp}$
